# Differential effects of lifestyle activities on disability incidence based on neighborhood amenities

**DOI:** 10.1186/s12877-023-04170-z

**Published:** 2023-08-10

**Authors:** Osamu Katayama, Sangyoon Lee, Seongryu Bae, Keitaro Makino, Ippei Chiba, Kenji Harada, Yohei Shinkai, Hiroyuki Shimada

**Affiliations:** 1 Department of Preventive Gerontology, Center for Gerontology and Social Science, National Center for Geriatrics and Gerontology, 7-430 Morioka-cho, Obu City, 474-8511 Aichi Japan; 2 Japan Society for the Promotion of Science, Tokyo, Japan; 3 Columbia University Irving Medical Center, New York, USA; 4 Department of Health Care and Science, Dong-A University, Busan, Korea; 5 Tohoku Medical Megabank Organization (ToMMo), Tohoku University, Sendai, Japan

**Keywords:** Disability, Lifestyle activity, Older adults, Neighborhood amenity, Environment

## Abstract

**Background:**

This study examined the effect of neighborhood amenities on disability risk among community-dwelling older adults in Japan, based on lifestyle activities.

**Method:**

This was an observational prospective cohort study. Participants comprised 13,258 older adults from the National Center for Geriatrics and Gerontology–Study of Geriatric Syndromes. We calculated participants’ Walk Score using their home addresses and divided them into three groups: “car-dependent,” “somewhat walkable,” and “very walkable.” We then calculated the average value of lifestyle activities. We divided the neighborhood amenity groups into two groups, “fewer lifestyle activities” and “more lifestyle activities,” for a total of six groups. After identifying interactions between neighborhood amenities and lifestyle activities, Cox proportional hazard models to calculate hazard ratios for incident disability risk, based on neighborhood amenities and lifestyle activities.

**Results:**

An interaction occurred between neighborhood amenities and lifestyle activities (*p* < 0.05). Survival probabilities for incident disability based on lifestyle activities were estimated for each neighborhood amenity group: car-dependent, 1.62 (95% CI 1.07 to 2.46); somewhat walkable, 1.08 (95% CI 0.84 to 1.40); and very walkable, 1.05 (95% CI 0.87 to 1.27). Those with fewer lifestyle activities in the car-dependent group exhibited the highest risk of incident disability in the unadjusted and adjusted models.

**Conclusion:**

Given that the aging population is increasing steadily, considering older adults’ neighborhood amenities and lifestyle activities in their day-to-day lives can help clinicians to deliver more older adult-centered care. Incorporating the lifestyle activities and neighborhood amenities of older adults into care planning will lead to the design and development of integrated clinical and community screening programs.

## Background

Several developed countries have rapidly aging populations, with Japan’s aging population increasing the fastest. As of 2020, the number of Japanese older than 65 was 35.9 million—28.4% of the population and the highest proportion globally [1]. Accordingly, Japan will have the largest proportion of older adults worldwide by 2050, when 39.9% of the national population will be older than 65 years [1]. In developed nations facing an aging population, many of these older adults require care [1, 2]. The World Health Organization (WHO) redefined healthy aging in its first global report on aging and health, making recommendations on the interaction between an individual’s intrinsic capabilities and relevant environmental characteristics [3].

In a previous study, living in a disadvantaged neighborhood was associated with lower active life expectancy and a greater percentage of projected remaining life with disability [4]. Another study indicated that the neighborhood deprivation level and neighborhood socioeconomic disadvantage influence stroke incidence, higher 30-day mortality and, incident and mortality cases of lung cancer [5–7]. However, while lifestyle activity is important for preventing disability among the older adults [8, 9], these previous studies have not adequately examined the lifestyle activity status of older adults living in disadvantaged neighborhoods.

Walkability has been shown to increase both physical activity and social capital (defined as social networks and interactions that inspire trust and reciprocity among citizens) and decrease the prevalence of obesity and depression [10]. It has also been found that older adults’ proximity and accessibility to important resources are associated with social participation, and that their environment influences their health-related behaviors [11, 12]. Moreover, the promotion of such social participation may be important for disability prevention, and it has been found that attending community salons can reduce disability risk [13]. Additionally, the frequency of participation in such gatherings is reported to be predicted by the proximity to one’s home, and reduced ability to perform activities of daily living (ADLs) is associated with disability occurrence [13]. These previous studies suggest that neighborhood amenities may promote healthy behaviors among older adults and prevent disability. However, it is not entirely clear to what extent the effect on disability prevention varies with lifestyle activity practices in each neighborhood divided by neighborhood amenities.

Therefore, this study aimed to clarify whether the effects of lifestyle activity on disability incidence differ based on neighborhood amenities among community-dwelling older adults in Japan. It was hypothesized that an active lifestyle could reduce disability risk, even in residential areas with limited neighborhood amenities.

## Methods

### Participants, design, and setting

**Fig. 1:**
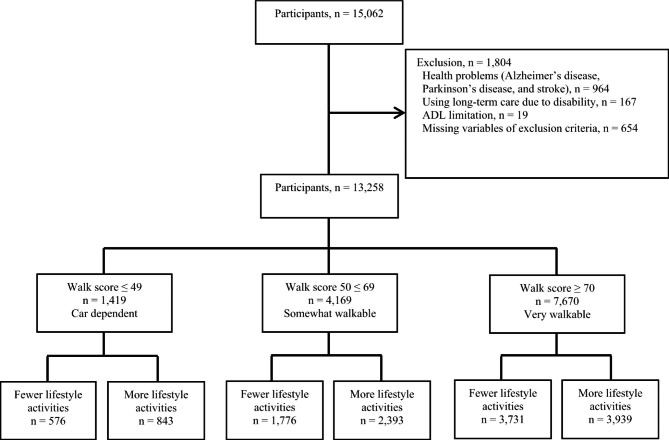
Flow diagram of sample selection

Participants were selected from the National Center for Geriatrics and Gerontology Study of Geriatric Syndromes (NCGG–SGS) for a study on health promotion for older adults in the neighboring cities of Obu, Nagoya, and Takahama, Japan. The NCGG–SGS is a cohort study aimed at establishing a geriatric syndrome screening system and validating evidence-based preventative interventions. Our inclusion criteria were as follows: all participants had to reside in the Obu, Nagoya or Takahama and be at least 70years or older in the Nagoya, 65years or older in Obu, and 60years or older in Takahama at the time of the study. Takahama’s age was set at 60years or older because many people in Japan reach retirement at 60 years of age, and the risk of health problems, such as frailty and disability, is thought to increase due to major lifestyle changes during this time. Obu, Nagoya, and Takahama City provided us with the address information of residents of older adults. We sent invitation letters to participants who lived in Obu, Nagoya, and Takahama City. Participants were not hospitalized, were not in residential care, were not certified by the long-term care insurance (LTCI) system as having functional disability, or were not participating in another study. Our inclusion criteria were as follows: a total of 15,062 community-dwelling older adults (Obu: 5628; Nagoya: 5257; Takahama: 4177) participated in assessments, including face-to-face interviews and physical and cognitive function measures. Exclusion criteria were as follows: (1) health problems such as dementia, Parkinson’s disease, or stroke (*n* = 964), based on information obtained through face-to-face interviews with participants by qualified nursing staff members, who complete a questionnaire about the names of diseases that have been previously diagnosed by a medical doctor; (2) need for support or care—as certified by the Japanese public long-term care insurance (LTCI) system—because of disability (*n* = 167); (3) disability affecting basic ADLs (*n* = 19); and (4) responses with missing variables (*n* = 654). Of the initial 15,062 participants, 1,804 were excluded based on these criteria. The final analysis included data from 13,258 older adults (7,033 women; mean age: 73.1 years, standard deviation [SD] = ± 5.9; age range: 60–97 years). The study was conducted according to the Declaration of Helsinki. All participants provided written informed consent before being included in the study. The study protocol was approved by the Ethics Committee of the National Center for Geriatrics and Gerontology (No. 1440–5) (Fig. 1).

### Measurement of neighborhood amenities

We used Walk Score™ (Front Seat Management, LLC, Seattle, WA) [14] to measure neighborhood amenities. The Walk Score is a publicly available website [15]. It has been found to be valid and reliable for estimating access to amenities within a comfortable walking distance. Walk Score identifies neighborhood amenities and calculates a walkability score [14] in one of 13 categories. The Walk Score calculation uses data provided by the Google™ AJAX search application program interface [16] and a geography-based algorithm. Because physical activity and social participation among older adults have been reported to be associated with neighborhood walkability [17, 18], we used walk scores as a surrogate marker for neighborhood amenities. Walk Score was based on the distance to 13 amenity categories: grocery stores, coffee shops, restaurants, bars, movie theatres, schools, parks, libraries, book stores, fitness centers, drug stores, hardware stores, and clothing/music stores [14]. Each category was weighted equally and points were calculated and normalized to yield a score of 0–100 (0: almost all errands require a car, 100: daily errands do not require a car), based on the distance from a specific address to local amenities [14]. Walk Score has been shown to be related to locally-derived and -built environment indices [15, 19, 20]. Walk scores were calculated using participants’ home addresses and divided into three groups according to predefined criteria and previous studies based on their distance from amenities [14, 21]: “car-dependent” (almost all errands require a car, but there are some amenities within walking distance); score: 0–49, “somewhat walkable” (some amenities within walking distance); 50–69, and “very walkable” (most errands can be accomplished on foot); 70–100.

### Measurement of lifestyle activities

Questionnaire items were adapted from the Japan Science and Technology Agency Index of Competence [22], Kihon Checklist [23], and NCGG–SGS [24]. From these questions, 10 activities that are performed outdoors and that appeared to be relevant to neighborhood amenities were selected based on the recommendations of gerontology experts. This study included physical, cognitive, and social activities, as previous studies have shown physical [25], cognitive [26], and social frailty [27] to be factors in the development of disability. Responses about these factors were collected using the following questionnaire items: (1) “Do you go out by bus or train by yourself? (go out by bus or a train)” (this question addressed whether participants used public transport for any reason); (2) “Do you go shopping for daily necessities by yourself? (go shopping)”; (3)“Do you manage your deposits and savings at the bank? (manage deposits)”; (4) “Do you sometimes visit your friends? (visit friends)”; (5) “Do you turn to your family or friends for advice? (turn to for advice)”; (6) “Do you go out at least once a week? (go out at least once a week)”; (7) “Do you normally walk continuously for 15 minutes? (walk continuously for 15 minutes)”; (8) “Do you have a paid job? (job)”; (9) “Do you drive a car? (drive a car)”; and (10) “Do you work as an officer or manager in your community? (officer or manager).” Participants were asked to answer “yes” or “no” to whether they had performed these activities during the past month. Additionally, the number of 10 lifestyle activities implemented by all participants was calculated, and to facilitate interpretation of the results, the participants were operationally classified into two groups, “fewer lifestyle activities” and “more lifestyle activities”. Since the number of lifestyle activities was not normally distributed, the median value was used to classify the two groups. We then divided the three neighborhood amenity groups into two groups each, “fewer lifestyle activities” and “more lifestyle activities,” for a total of six groups.

### Disability determination

All participants were tracked monthly for the new incidence of LTCI certification, as recorded by the Japanese LTCI system, during the two years after baseline assessment, which is managed by each municipal government. The LTCI system classifies a person as “Support Level 1 or 2” to indicate need for assistance to support ADLs or “Care Levels 1 through 5” to indicate a need for continuous care [28]. This study defined disability as any LTCI certification level; we defined disability onset as the point at which a participant received LTCI certification.

### Potential confounding factors

Demographic variables and chronic diseases are associated with disability in older adults. Crude and adjusted Cox proportional hazard models included the following covariates for participants: age, gender, Mini-Mental State Examination (MMSE) [29] score, number of medications, chronic diseases (hypertension, heart disease, diabetes, and depression), depression, 15-item Geriatric Depression Scale (GDS) [30], walking speed, and handgrip strength. The presence of hypertension, heart disease, and diabetes was also entered into the models, based on self-report data.

To measure walking speed to assess physical function, participants were asked to walk a predetermined distance of 2.4 m at a comfortable speed (m/s). We used two markers to indicate the start and end of the path. Participants were instructed to start walking 2 m before the starting marker and to continue walking another 2 m from the end point to ensure a constant walking speed [31]. Walking speed was measured five times and the average value was taken as representative. In addition, handgrip strength (kg) was measured using a Smedley-type handheld dynamometer (GRIP-D; Takei Scientific Instruments Co., Ltd., Niigata, Japan). Handgrip strength measurements were recorded for each participant’s dominant hand in a standing position with elbows extended, using the same device [31].

### Statistical analysis

One-way ANOVA and Pearson’s chi-squared tests were used to compare variables among neighborhood amenity groups. Adjusted standardized residuals > 1.96 indicated *p* < 0.05. The cumulative incidence of disability during follow-up was calculated for “fewer lifestyle activities” and “more lifestyle activities” for each of the three groups (i.e., “car dependent”, “somewhat walkable”, and “very walkable”) according to Kaplan–Meier curves. Intergroup differences were estimated using log-rank tests. The p-values for interactions between neighborhood amenities and lifestyle activities were calculated using a multivariate Cox proportional hazard model. Crude and adjusted Cox proportional hazard models were constructed to calculate hazard ratios with 95% confidence intervals (CI) for incident disability risk [32, 33]. The significance level was set at p < 0.05. All analyses were performed using IBM SPSS version 28.0 (IBM Corp., Armonk, NY, USA).

## Results

Of the study population, the car-dependent, somewhat walkable, and very walkable groups accounted for 1,419 (10.7%), 4,169 (31.4%), and 7,670 (57.9%) participants, respectively. Descriptive statistics for all variables, based on neighborhood amenities status, are shown in Table 1. Significant differences were observed among the three groups concerning age, MMSE score, number of medications, walking speed, handgrip strength, and number of lifestyle activities (*p* < 0.001). According to the Kaplan–Meier analysis and log-rank tests in each neighborhood amenity group, the car-dependent, somewhat walkable, and very walkable groups revealed significant intergroup differences among disability incidence rates (*p* < 0.001).

**Table 1:** Demographic characteristics of older adults from each neighborhood amenity group

	Total n = 13,258	Car-dependent n = 1419 (10.7)	Somewhat Walkable n = 4169 (31.4)	Very Walkable n = 7670 (57.9)	*p* value	η^2^	Post-hoc
Age (years)	73.11 ± 5.94	71.78 ± 5.74	72.14 ± 6.02	73.88 ± 5.82	< 0.001^*^	0.024	Car, Somewhat < Very
Women, number (%)	7033 (53.0)	740 (52.1)	2163 (51.9)	4130 (53.8)	0.096^†^		
MMSE score	26.42 ± 2.69	26.25 ± 2.80	26.55 ± 2.68	26.38 ± 2.67	< 0.001^*^	0.001	Car, Very < Somewhat
Medication, number	2.64 ± 2.56	2.24 ± 2.29	2.32 ± 2.34	2.89 ± 2.69	< 0.001^*^	0.013	Car, Somewhat < Very
Hypertension, yes	6121 (46.2)	680 (47.9)	1871 (44.9)	3570 (46.6)	0.084^†^		
Heart disease, yes	2206 (16.7)	231 (16.3)	644 (15.5)^§^	1331 (17.4)^‡^	0.026^†^		
Diabetes, yes	1723 (13.0)	200 (14.1)	553 (13.3)	6697 (12.7)	0.275^†^		
Depression, yes	388 (2.9)	39 (2.7)	109 (2.6)	240 (3.1)	0.260^†^		
GDS score	2.87 ± 2.65	2.88 ± 2.57	2.82 ± 2.61	2.89 ± 2.68	0.445^*^	< 0.001	
Walking speed, m/sec	1.13 ± 0.23	1.17 ± 0.22	1.15 ± 0.22	1.11 ± 0.23	< 0.001^*^	0.011	Very < Somewhat < Car
Grip Strength, kg	26.89 ± 7.78	27.20 ± 7.65	27.45 ± 7.92	26.54 ± 7.71	< 0.001^*^	0.003	Very < Car, Somewhat
Lifestyle activities, number	7.48 ± 1.48	7.66 ± 1.49	7.58 ± 1.48	7.40 ± 1.47	< 0.001^*^	0.005	Very < Car, Somewhat

*P-values reported from one-way ANOVA. Significant difference obtained by Tukey post-hoc test

†P-values obtained by Pearson’s chi-squared test. ‡Statistically significant association by adjusted standardized residual > 1.96 (p < 0.05). §Statistically significant association by adjusted standardized residual < − 1.96 (p < 0.05)

MMSE: Mini-Mental State Examination; GDS: 15-item Geriatric Depression Scale; Car: car-dependent; Somewhat: somewhat walkable; Very: very walkable

The characteristics of all six groups—when combining neighborhood amenity status and lifestyle activities—were assessed. Each neighborhood amenity group was divided into two groups based on participants having more than or fewer than eight lifestyle activities. The fewer and more lifestyle activities groups respectively accounted for 576 (4.3%) and 843 (6.4%) participants in the car-dependent group; 1,776 (13.4%) and 2,393 (18.0%) in the somewhat walkable group; and 3,731 (28.1%) and 3,939 (29.7%) in the very walkable group. Descriptive statistics for all variables for participant characteristics grouped by combining neighborhood amenities status and lifestyle activities are shown in Table 2. Significant differences were observed among the six groups concerning age, gender, MMSE score, number of medications, hypertension, heart disease, depression, GDS score, walking speed, and handgrip strength (*p* < 0.01).

**Table 2:** Demographic characteristics of the six groups combining neighborhood amenities status and lifestyle activities

	Total	Car-dependent			Somewhat walkable			Very walkable				
		**Fewer lifestyle activities**	**More lifestyle activities**		**Fewer lifestyle activities**	**More lifestyle activities**		**Fewer lifestyle activities**	**More lifestyle activities**	***p*** **value**	**η** ^**2**^	**Post-hoc**
Prevalence, number (%)	13,258	576 (4.3)	843 (6.4)		1776 (13.4)	2393 (18.0)		3731 (28.1)	3939 (29.7)			
Age (year)	73.11 ± 5.94	72.89 ± 6.25	71.02 ± 5.24		73.14 ± 6.63	71.39 ± 5.41		74.92 ± 6.00	72.90 ± 5.46	< 0.001^*^	0.050	CM < CF, SF, VF, VM; SM < CF, SF, VF, VM; CF, SF, VM < VF
Women, number (%)	7033 (53.0)	331 (57.5)^‡^	409 (48.5) ^§^		1020 (57.4)^‡^	1143 (47.8)^§^		2263 (60.7)^‡^	1867 (47.4)^§^	< 0.001^†^		
MMSE score	26.42 ± 2.69	25.91 ± 3.06	26.47 ± 2.58		26.25 ± 2.87	26.76 ± 2.51		26.07 ± 2.83	26.68 ± 2.47	< 0.001^*^	0.012	CF, VF < CM, SM, VM; SF < SM, VM
Medication, number	2.64 ± 2.56	2.53 ± 2.37	2.04 ± 2.21		2.67 ± 2.47	2.07 ± 2.20		3.24 ± 2.86	2.56 ± 2.48	< 0.001^*^	0.028	CF, SF, VM < VF; CM, SM < CF, SF, VF, VM
Hypertension, yes	6121 (46.2)	305 (53.0) ^‡^	375 (44.5)		858 (48.3) ^‡^	1013 (42.3)^§^		1806 (48.4)^‡^	1764 (44.8)^§^	< 0.001^†^		
Heart disease, yes	2206 (16.7)	82 (14.3)	149 (17.7)		293 (16.5)	351 (14.7)^§^		692 (18.6)^‡^	639 (16.2)^§^	0.001^†^		
Diabetes, yes	1723 (13.0)	91 (15.8)	109 (12.9)		236 (13.3)	317 (13.2)		502 (13.5)	468 (11.9)^d^	0.098^†^		
Depression, yes	388 (2.9)	13 (2.3)	26 (3.1)		64 (3.6)	45 (1.9)^§^		129 (3.5)^‡^	111 (2.8)^§^	0.005^†^		
GDS score	2.87 ± 2.65	3.80 ± 2.74	2.24 ± 2.24		3.63 ± 2.83	2.23 ± 2.26		3.62 ± 2.90	2.20 ± 2.25	< 0.001^*^	0.072	CM, SM, VM < CF, SF, VF
Walking speed, m/sec	1.13 ± 0.23	1.11 ± 0.24	1.21 ± 0.20		1.09 ± 0.23	1.18 ± 0.21		1.06 ± 0.23	1.15 ± 0.21	< 0.001^*^	0.050	CF, SF < CM, SM, VM; SM < CM; VM < SM; VF < CF, CM, SF, SM, VM
Grip Strength, kg	26.89 ± 7.78	25.83 ± 7.48	28.12 ± 7.63		25.98 ± 7.78	28.54 ± 7.84		24.91 ± 7.45	28.06 ± 7.64	< 0.001^*^	0.037	CF, SF < CM, SM, VM; VF < CM, SF, SM, VM

^*^P-values reported from one-way ANOVA. Significant difference obtained by Tukey post-hoc test

^†^P-values obtained by Pearson’s chi-squared test. ^‡^Statistically significant association by adjusted standardized residual > 1.96 (*p* < 0.05). ^§^Statistically significant association by adjusted standardized residual < − 1.96 (*p* < 0.05)

MMSE: Mini-Mental State Examination; GDS: 15-item Geriatric Depression Scale; CF: Car-dependent group with fewer lifestyle activities; CM: Car-dependent group with more lifestyle activities; SF: Somewhat Walkable group with fewer lifestyle activities; SM: Somewhat Walkable group with more lifestyle activities; VF: Very Walkable group with fewer lifestyle activities; VM: Very Walkable group with more lifestyle activities

**Fig. 2:**
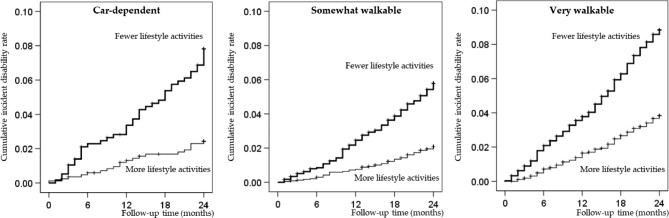
Kaplan–Meier estimates of cumulative incidence of disability according to lifestyle activity status

In this study, 672 (5.1%) of participants developed disability during the follow-up period, with a mean follow-up of 36.5 months (SD = 12.2 months) from baseline. Onset probabilities for incident disability were determined using the Cox proportional hazard risk analysis. An interaction was observed between neighborhood amenities and lifestyle activities (*p* < 0.05). Figure 2 shows the probability of independence according to the Kaplan–Meier analysis in each group; the “fewer lifestyle activities” group showed a higher rate of disability onset. Log-rank tests revealed significant intergroup differences among disability incidence rates (*p* < 0.01).

Table 3 shows the unadjusted and adjusted survival probabilities for incident disability using the Cox proportional hazard risk analysis in each neighborhood amenity group. Survival probabilities for incident disability in the fewer lifestyle activities group were estimated for each neighborhood amenity group, as follows: car-dependent group, 1.62 (95% CI 1.07–2.46); somewhat walkable group, 1.08 (95% CI 0.84–1.40); and very walkable group, 1.05 (95% CI 0.87–1.27). The fewer lifestyle activities group in the car-dependent group exhibited a higher hazard ratio of incident disability in both the crude and adjusted models.

**Table 3:** Cox regression analysis of the relationships between lifestyle activities and incident disability in each neighborhood amenity group

**Car-dependent**	**Number of Participants**	**Incident Disability Rate**	**Crude Model**				**Adjusted model**		
			**HR**	**95% CI**	***P***		**HR**	**95% CI**	***P***
More lifestyle activities	843	20 (2.4%)	1.00				1.00		
Fewer lifestyle activities	576	43 (7.5%)	2.75	1.92―3.92	< 0.001		1.62	1.07―2.46	0.022
**Somewhat walkable**	**Number of ** **Participants**	**Incident ** **Disability Rate**	**Crude Model**				**Adjusted model**		
			**HR**	**95% CI**	***P***		**HR**	**95% CI**	***P***
More lifestyle activities	2393	49(2.0%)	1.00				1.00		
Fewer lifestyle activities	1776	99(5.6%)	2.37	1.90―2.96	< 0.001		1.08	0.84―1.40	0.560
**Very walkable**	**Number of ** **Participants**	**Incident ** **Disability Rate**	**Crude Model**				**Adjusted model**		
			**HR**	**95% CI**	***P***		**HR**	**95% CI**	***P***
More lifestyle activities	3939	147(3.7%)	1.00				1.00		
Fewer lifestyle activities	3731	314(8.4%)	2.20	1.87―2.60	< 0.001		1.05	0.87―1.27	0.597

Adjusted model is adjusted for the covariates in age, sex, MMSE score, medications, hypertension, heart disease, diabetes, depression, GDS score, walking speed, and grip strength

HR: Hazard ratio

## Discussion

This study aimed to clarify the difference in the effects of lifestyle activities on disability incidence based on neighborhood amenities among community-dwelling older adults in Japan. In support of the initial hypothesis, the results indicated that participants who reported engaging in many lifestyle activities also showed a reduced risk of developing a disability, even in residential areas with limited nearby neighborhood amenities.

It has been previously reported that reduced ability to perform ADLs, physical, cognitive, and social frailty, and motoric cognitive risk syndrome are associated with disability occurrence [13, 25–27, 34]. Additionally, participation in meetings such as salon activities that promote social interaction reduces disability risk [13]. However, few studies have considered both daily living activities and neighborhood amenities. This study sought participants’ responses about their lifestyle activities related to outdoor activities (e.g., going out by bus or a train, shopping, going out at least once a week, and walking continuously for 15 min), cognitive activities (e.g., managing deposits and driving a car), and social activities (e.g., visiting friends, seeking advice from others, employment, and being an officer or manager). In this study, as in these previous studies, low engagement in these lifestyle activities was found to be associated with increased disability risk. A novel finding of our study is that the implementation of lifestyle activities has a strong influence on the occurrence of disability among older adults living in neighborhoods with poor neighborhood amenities. Neighborhood amenities may have a protective effect on disability incidence in the somewhat walkable and very walkable groups. Differences in the incidence of disability might be the socioeconomic structures of the neighborhood and lifestyle factors shaped over the life course by neighborhood amenities [13, 35]. This finding suggests that neighborhood amenities may also have major implications for health equity in affluent/safe versus non-affluent/unsafe neighborhoods—an area worthy of future investigation.

In a previous study, the number of community salons participants attended was strongly predicted by the number of community salons within a 350-meter radius of each participant’s home [13]. Thus, one’s physical and social environment may influence engagement in lifestyle activity. Neighborhood characteristics—such as amenities and whether the area is urban—have often been associated with physical activity levels among older adults [36–39]. Walk Score, used as an indicator of neighborhood amenities in this study, helped calculate a walkability score [14] based on the distance to amenities in 13 categories. Thus, the neighborhood amenities group may reflect the number of places around a participant’s home that promote social interaction. In the car-dependent group, there may be few such locations around participants’ homes, which may have been a factor in the fewer lifestyle activities group’s higher risk of developing a disability. Thus, the concomitant decline in lifestyle activities and neighborhood amenities suggested that the fewer lifestyle activities group was at a higher hazard ratio of developing a disability. However, this study failed to consider that there is a difference between objectively-measured and perceived neighborhood amenities; this limitation should be addressed in the future. Future studies should also consider the research on perceived neighborhood amenities for each participant.

This study’s strengths include the large sample size and an operationalized assessment to identify neighborhood amenities, lifestyle activity, and disability risk. However, some limitations should be considered. First, our study did not use random sampling for data collection; hence, under-reporting of the disability incidence rate among older adults is possible. The participants were all capable of accessing health check-ups from their homes, implying that people with various other conditions were excluded. Second, the monthly incidence of new LTCI certifications, as recorded by the Japanese LTCI system and measured by the municipalities, was tracked. Of the participants in this study, 126 died and 51 were terminated because they moved away. It cannot be ruled out that these factors may have been competing risks for disability. In addition, participants may have been taking different medications at baseline and at follow-up. It is also possible that participants may have been hospitalized for acute diseases that could have put them at risk for developing disability. Future studies should consider these variables. Third, this study failed to address other covariates related to health variables (e.g., smoking or alcohol use) that could also affect cumulative age-related changes; therefore, future studies should include such variables. Fourth, because we did not investigate whether participants used nearby amenities, we were unable to determine the effect of amenity use on disability incidence. Finally, although this study focused on neighborhood amenities as an environmental factor, previous studies show that mobility disability results from the interaction of individual and environmental factors; [25, 40–42] therefore, we believe that future research should include other environmental characteristics using multi-level analysis, which allows for the hierarchy of the data.

Despite these limitations, our findings are significant in that we found that participants who engaged in fewer lifestyle activities exhibited the highest risk of incident disability. Growing evidence suggests that the physical and social environment in which individuals live may have a substantial influence on their physical and mental health [43–46]. These neighborhood effects may be particularly important for older adults, who are likely to spend more time in their residential neighborhood than younger people, and may be more sensitive to neighborhood characteristics, such as safety and physical access [47, 48]. Clinician’s role in disability prevention is to incorporate into the plan of care social health determinants [49], including activity status and environmental factors.

The clinical significance of this study is that understanding the implementation of lifestyle activities, in addition to neighborhood amenities, may be important in preventing disability among older adults. Characterizing individuals with lower engagement in lifestyle activities and identifying older adults who are amenable to preventative strategies could help clinicians to develop personalized approaches to facilitate participation in lifestyle activities. In particular, it is important to promote lifestyle activities in regions with limited neighborhood amenities. This study did not measure the intensity and duration of each lifestyle activity. For researchers, this is an area worthy of additional investigation. Policy-makers could use the results of this study to consider ways of preventing disability among older adults, such as designing amenities that would enhance the neighborhood amenities of older adults.

## Conclusions

This study’s findings indicate that the effects of lifestyle activities on disability incidence differ, based on neighborhood amenities among community-dwelling older adults. Given that the aging population is increasing steadily—including individuals who need long-term care—and that this population has a strong association with numerous adverse health outcomes, considering older adults’ neighborhood amenities and lifestyle activities in their day-to-day lives can help clinicians to deliver more older adult-centered care. This could lead to better outcomes in the primary prevention of disease. For clinicians to prevent disability among their own clients, as well as in their clients’ community, incorporating the personal (lifestyle activities) and environmental (neighborhood amenities) factors of older adults identified by this study into care planning may facilitate the design and development of integrated clinical and community screening programs.

## Data Availability

The datasets used and/or analyzed during the present study are available from the corresponding author on reasonable request.

## Abbreviations

LTCI: Long-term care insurance
NCGG–SGS: National Center for Geriatrics and Gerontology Study of Geriatric Syndromes
MMSE: Mini-Mental State Examination
GDS: Geriatric Depression Scale
ADL: Activities of Daily Living
CI: Confidence Interval
